# Supplementary material for: Purines enrich root-associated Pseudomonas and improve wild soybean growth under salt stress
Source: Nat Commun. 2024 Apr 25;15:3520. doi: 10.1038/s41467-024-47773-9 (PMC11045775; doi:10.1038/s41467-024-47773-9)
Supplement: Supplementary file 1 — Supplementary Information [file 41467_2024_47773_MOESM1_ESM.pdf]

Supplementary for

Purines enrich root-associated *Pseudomonas* and improve wild soybean growth under salt stress

Yanfen Zheng[1,4], Xuwen Cao[2,4], Yanan Zhou[1,3], Siqi Ma[1], Youqiang Wang[1], Zhe Li[1], Donglin Zhao[1], Yanzhe Yang[1], Han Zhang[1], Chen Meng[1], Zhihong Xie[3], Xiaona Sui[1], Kangwen Xu[1], Yiqiang Li[1], Cheng-Sheng Zhang[1*]

[1]Marine Agriculture Research Center, Tobacco Research Institute of Chinese Academy of Agricultural Sciences, Qingdao, 266101, China

[2]Institute of Marine Science and Technology, Shandong University, Qingdao, 266200, China

[3]National Engineering Laboratory for Efficient Utilization of Soil and Fertilizer Resources, College of Resources and Environment of Shandong Agricultural University, Taian, 271018, China

[4]These authors contributed equally: Yanfen Zheng, Xuwen Cao.

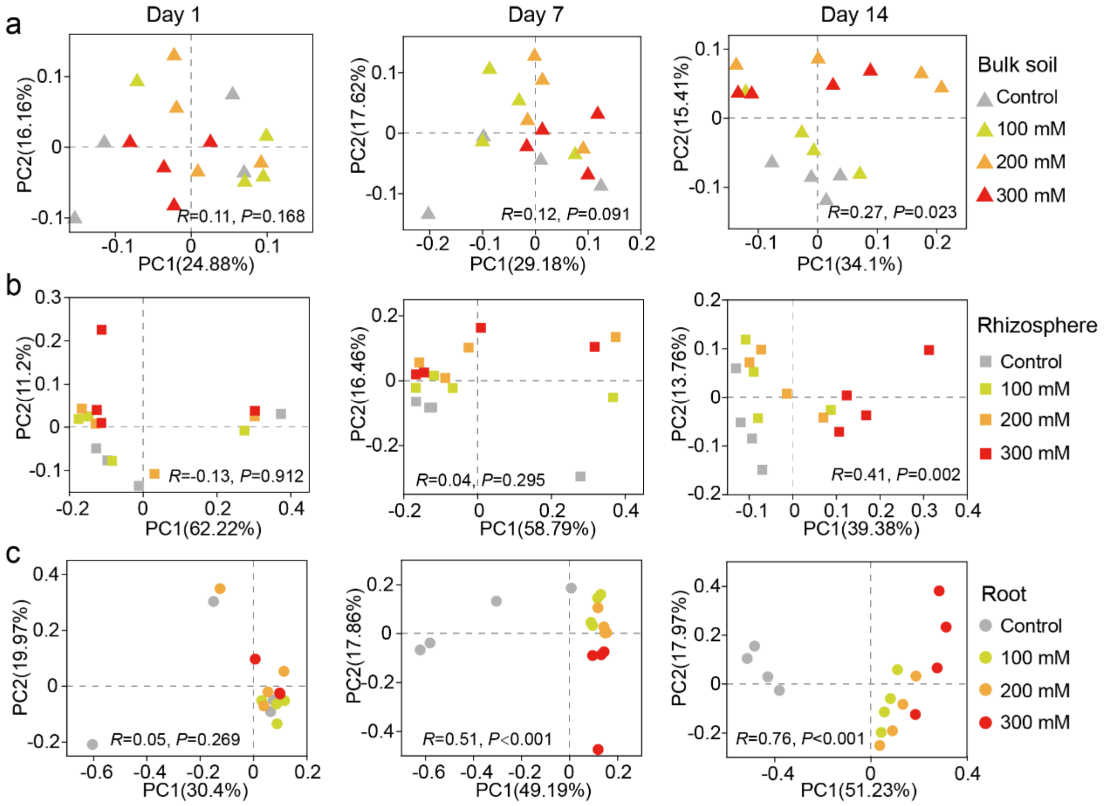

Supplementary Figure 1. Principal coordinates analysis (PCoA) with Bray-Curtis dissimilarity matrix performed on plant compartements and time points. a PCoA of bacterial community in bulk soil at OTU level; **b** PCoA of microbial comminity in rhizosphere soil at OTU level; **c** PCoA of microbial comminity in root at OTU level. Statistical analysis is performed using ANOSIM (analysis of similarities). Source data are provided as a Source Data file.

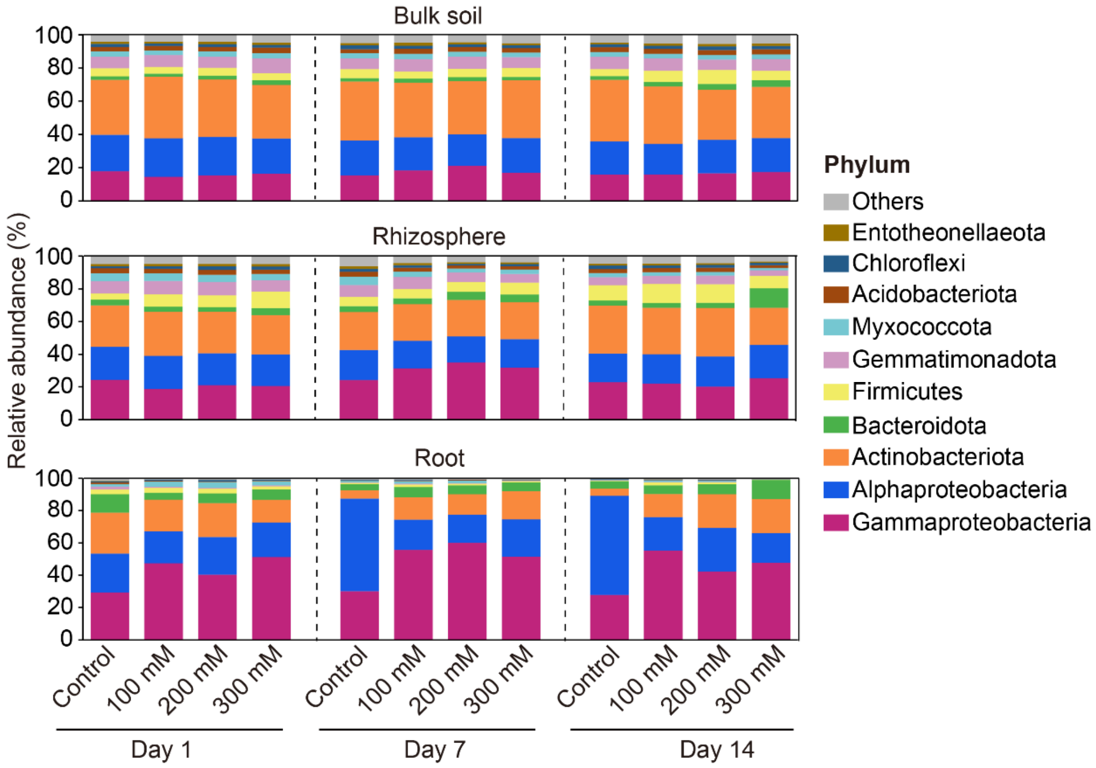

Supplementary Figure 2. Percent relative abundance of the top 10 most abundant phyla across the whole dataset. Proteobacteria are shown at the class level. Source data are provided as a Source Data file.

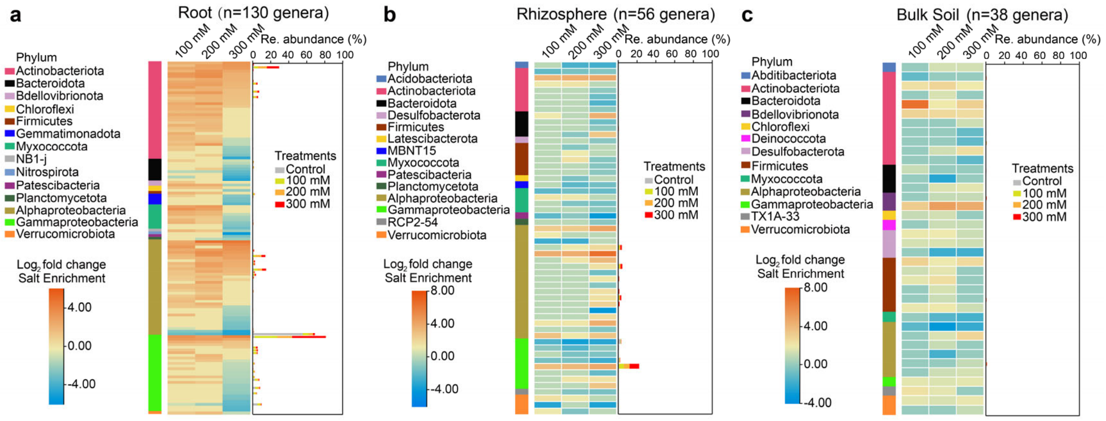

Supplementary Figure 3. Heatmap of enriched or depleted genera in salt treatments compared with control group. a A total of 130 genera showing significant change in root. **b** A total of 56 genera showing significant change in rhizosphere soil. **c** A total of 38 genera showing significant change in bulk soil. The genus showing significant change (|$\log_2$ fold change| >1 and two-sided Student's *t*-test $P < 0.05$) in any salt treatment is considered as salt enriched or depleted taxon. The fold change (left) and relative abundance (right) of all changed genera are shown. The left-most bar in each panel represents the phylum of each genera belongs to (Proteobacteria are shown at the class level). Source data are provided as a Source Data file.

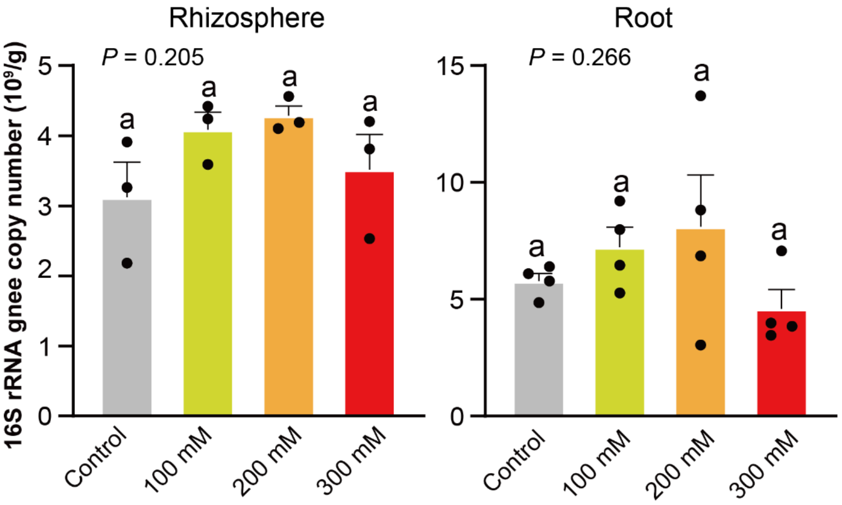

Supplementary Figure 4. The total bacterial abundance in control and salt-treated samples across rhizosphere and root compartments determined by qPCR. Values are means ± SEM (*n* = 3 rhizosphere soil samples; *n* = 4 root samples). Significance is determined using one-way ANOVA with correction by Tukey's HSD test. Source data are provided as a Source Data file.

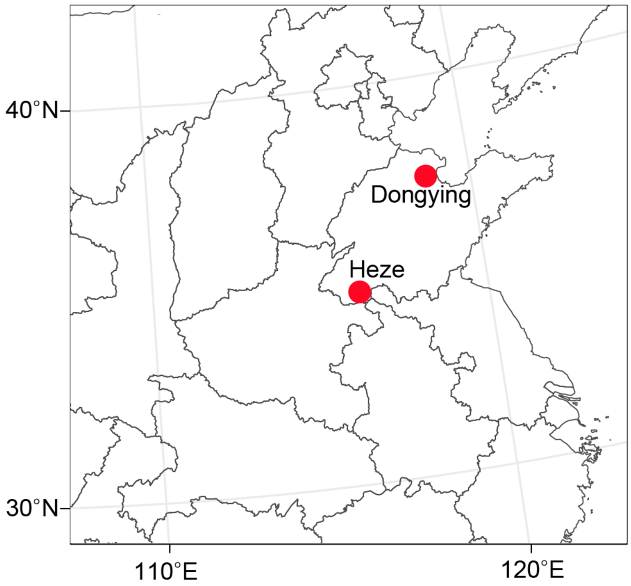

Supplementary Figure 5. Sampling sites of soil used in salt experiment to investigate *Pseudomonas* enrichment. Source data are provided as a Source Data file.

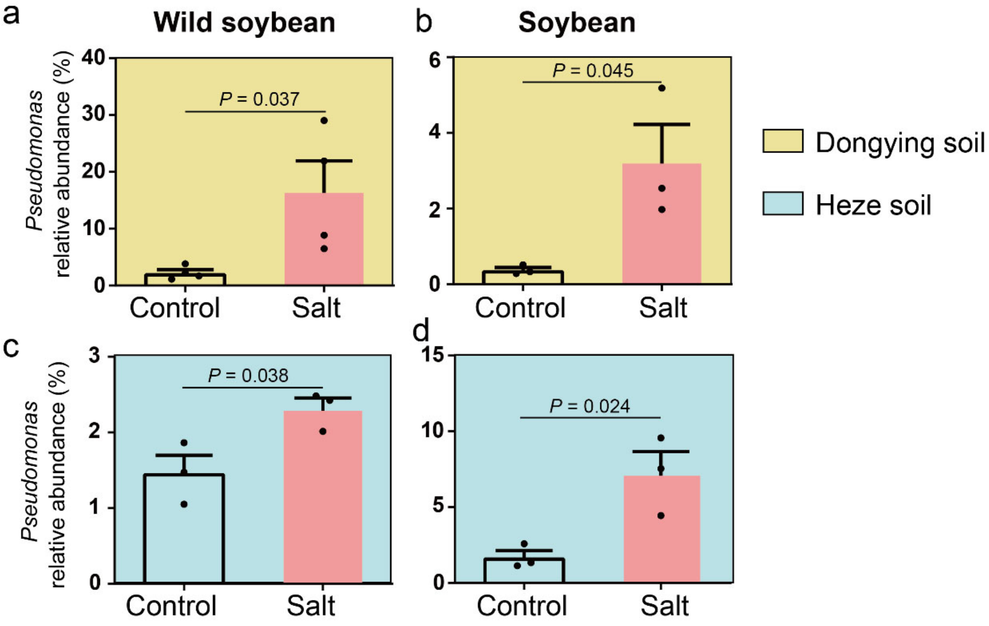

Supplementary Figure 6. The relative abundance of *Pseudomonas* in wild soybean and domesticated soybean roots within different soils. a, b The *Pseudomonas* abundance of wild soybean (**a**) and soybean (**b**) in Dongying soil based on 16S rRNA gene amplicon data. **c, d** The *Pseudomonas* abundance of wild soybean (**c**) and soybean (**d**) in Heze soil based on 16S rRNA gene amplicon data. Values are means ± SEM (n = 4 samples for figure **a**; n = 3 samples for figures **b**, **c**, **d**). P values are determined by two-sided Student's t-test. Source data are provided as a Source Data file.

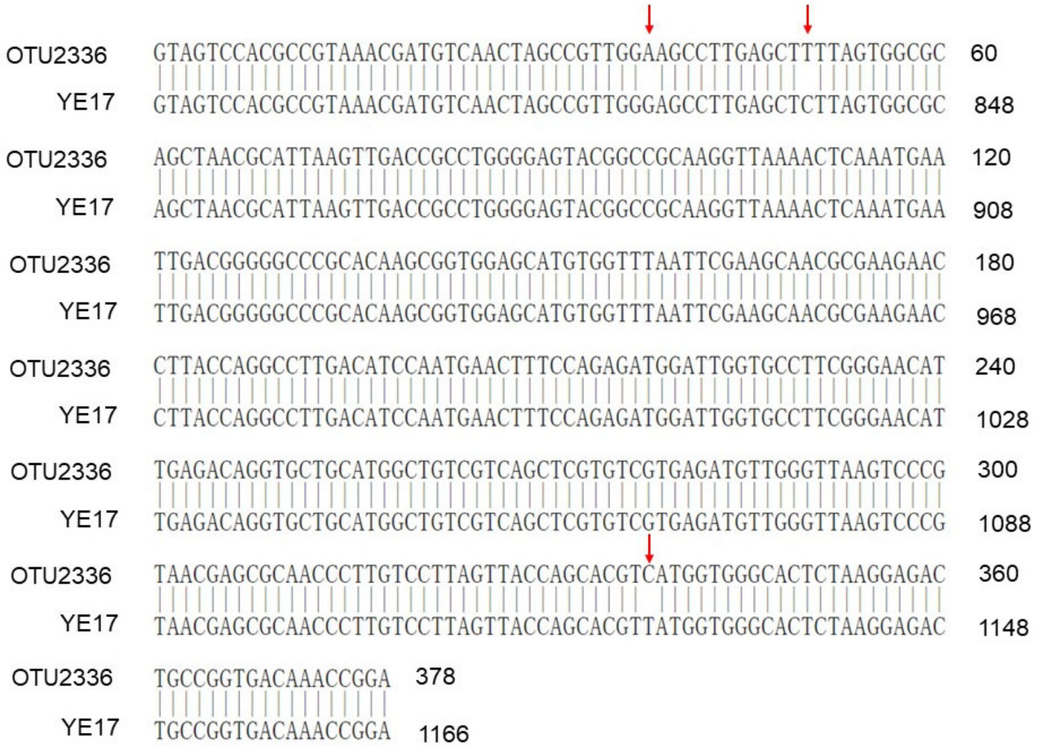

Supplementary Figure 7. Sequence alignments of the 16S rRNA gene sequence between strain YE17 and the salt-responsive OTU2336. Arrows indicate unmatched nucleotides. Strain YE17 showed 99.21% match to the *Pseudomonas* OTU2336. Source data are provided as a Source Data file.

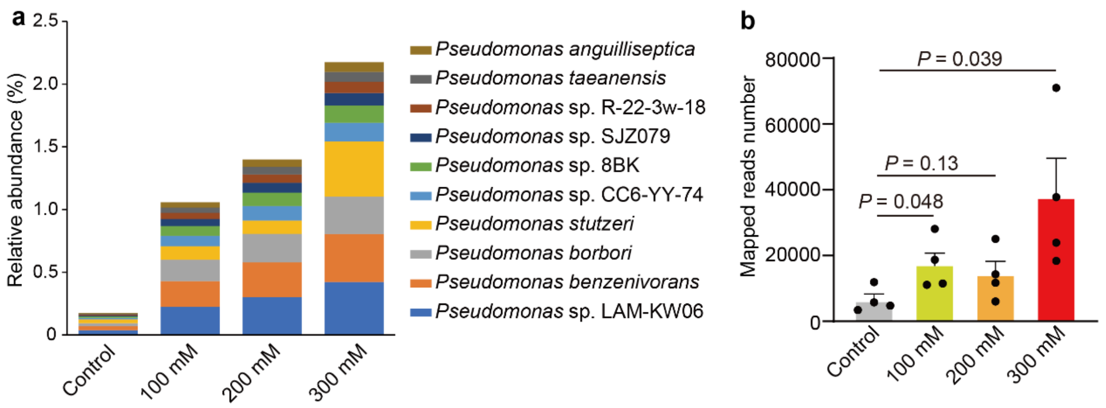

Supplementary Figure 8. The abundance of *P. stutzeri* determined by metagenomic data. a The top 10 most abundant *Pseudomonas* species. **b** The mapped reads number of strain XN05-1 genome to metagenomic data of different treatments. Values in panel **b** are means ± SEM (n = 4 rhizosphere soil samples). P values are determined by two-sided Student's t-test. Source data are provided as a Source Data file.

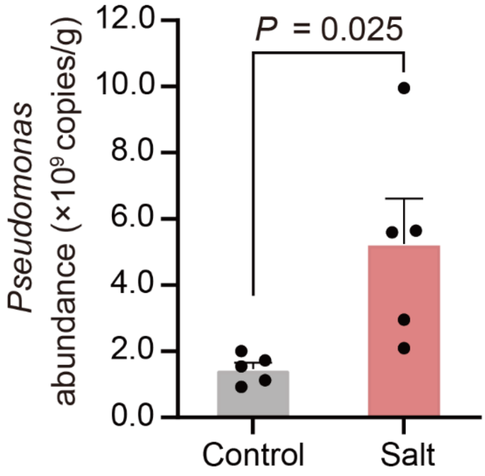

Supplementary Figure 9. *Pseudomonas* absolute abundance within the wild soybean root under salt and non-salt conditions after *Pseudomonas* inoculation. Values are means ± SEM (n = 5 biological replicates). P value is determined by two-sided Student's t-test. Source data are provided as a Source Data file.

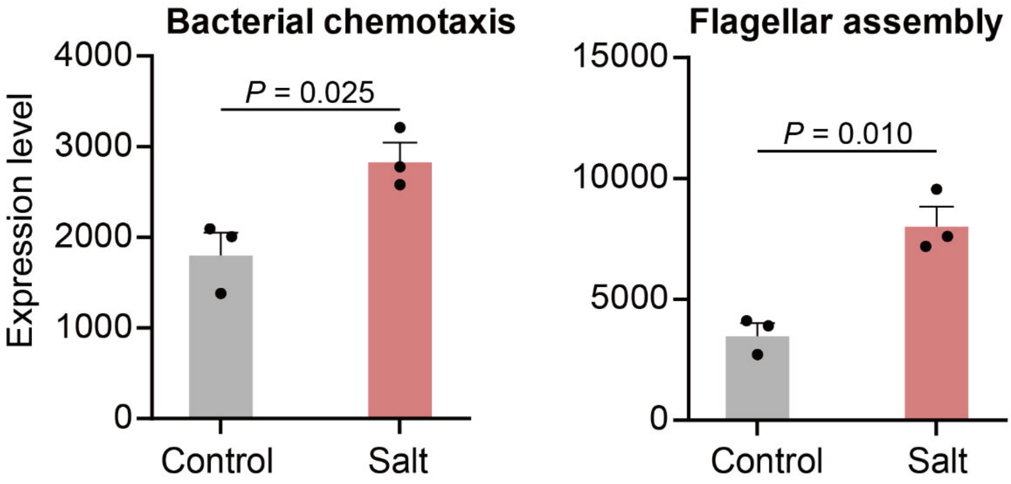

Supplementary Figure 10. The expression level of bacterial chemotaxis and flagella assembly subcategories in the control and salt-treated (200 mM NaCl) rhizospheres revealed by metatranscriptomic data. Each data is mean ± SEM (*n* = 3 rhizosphere soil samples). *P* values are determined by two-sided Student's *t*-test. Source data are provided as a Source Data file.

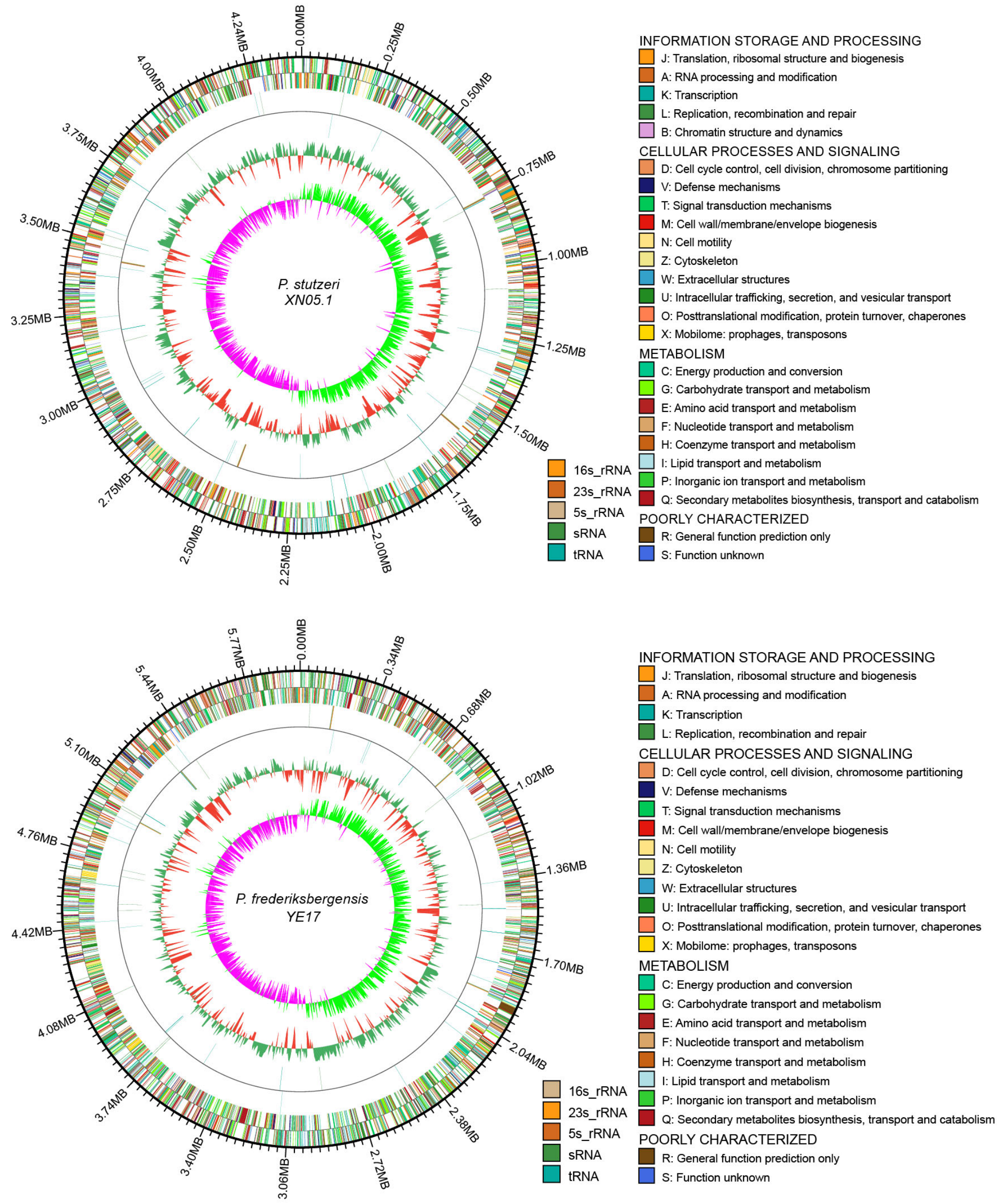

Supplementary Figure 11. Circular representation of strain XN05-1 and YE17 genomes. The outer scale is in mega bases (M). Circle 1 (from outside to inside): the marker of genome size. Circle 2 and 3: CDS with positive and negative chains; different colors represent different functional classifications. Circle 4 and 5: sRNA, tRNA, and rRNA with positive and negative chains. Circle 6: GC content; the inward red part indicates that the GC content in this region is lower than the genome-wide average GC content, and the outward green part is the opposite. Circle 7: the GC skew value; the algorithm is (G – C)/(G + C). The inward pink part indicates that the area contains less G than C, and the outward light green part is the opposite.

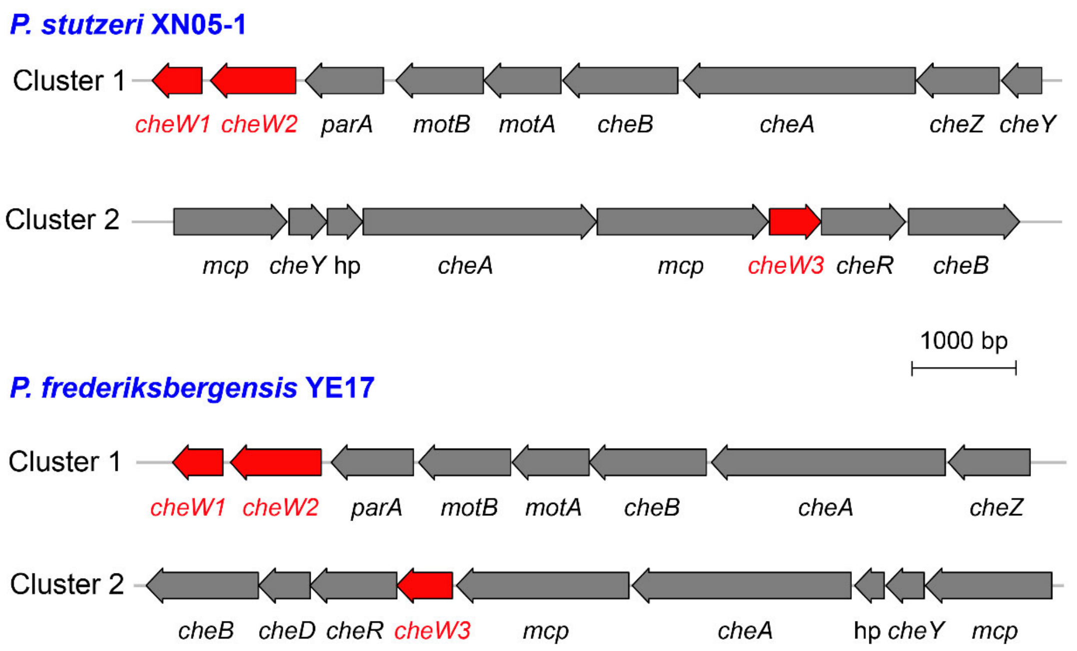

Supplementary Figure 12. Chemotaxis gene clusters in strain XN05-1 and YE17 genomes. Red arrows represent *cheW* used in gene knockout experiment. *parA*: chromosome partitioning protein; hp: hypothetical protein. Source data are provided as a Source Data file.

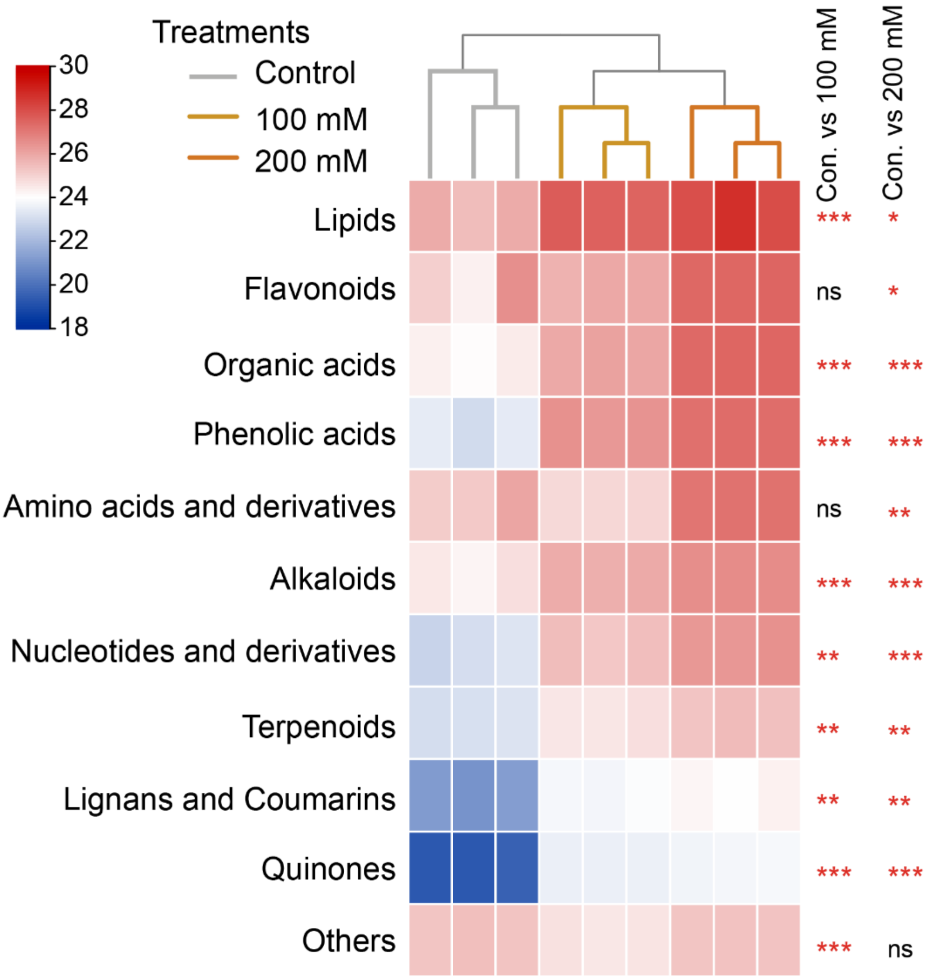

Supplementary Figure 13. Dynamic root exudates in two salt-treated and control wild soybean plants. Significance was determined with two-sided Student's *t*-test (****P*<0.001, ***P*<0.01, **P*<0.05, *P* values are shown in source data). Source data are provided as a Source Data file.

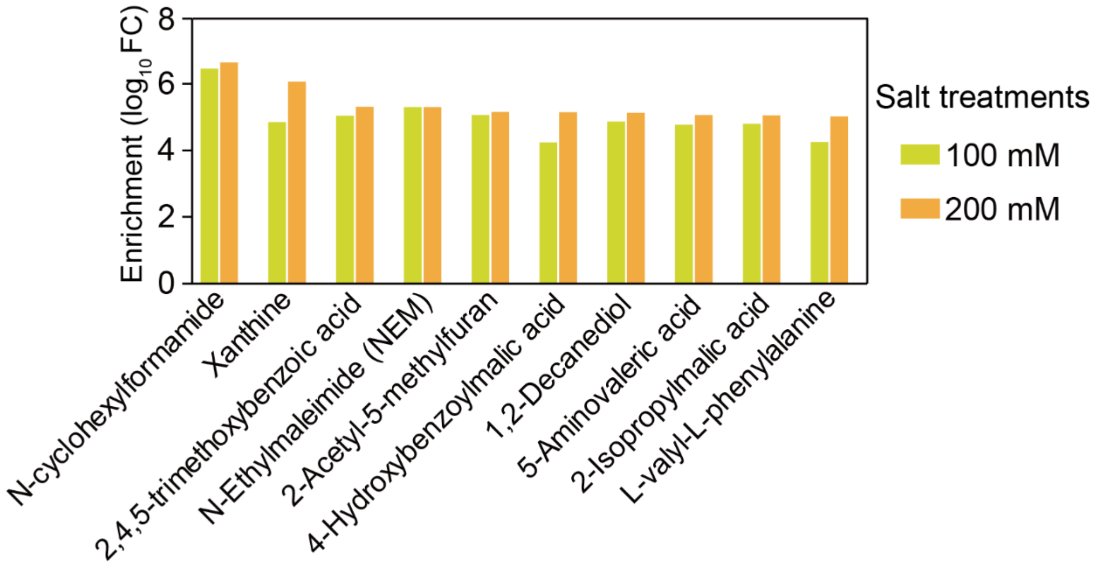

Supplementary Figure 14. The top 10 highest log_{10}-fold enrichment metabolites within salt treated root compared with control. Source data are provided as a Source Data file.

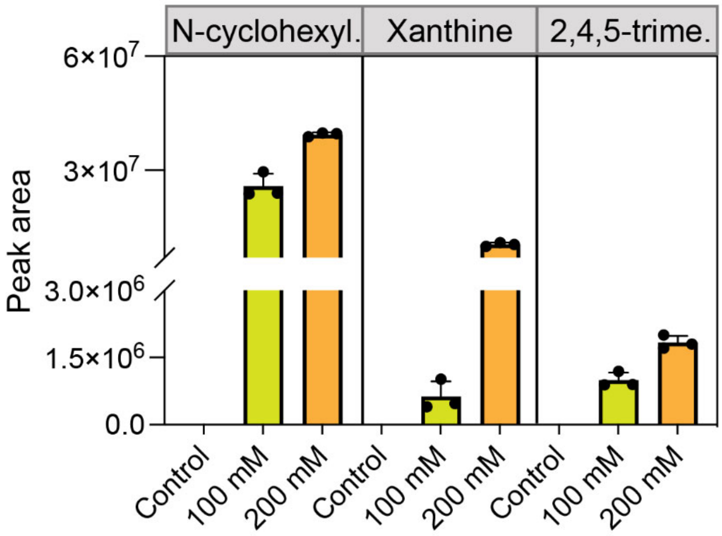

Supplementary Figure 15. The content of top three enriched compounds in salt treated samples according to metabolome sequencing. Each data is mean ± SEM (*n* = 3 biological replicates). Abbreviation: N-cyclohexyl., N-cyclohexylformamide; 2,4,5-trime., 2,4,5-trimethoxybenzoic acid. The contents of these compounds are hardly measured in the control group. Source data are provided as a Source Data file.

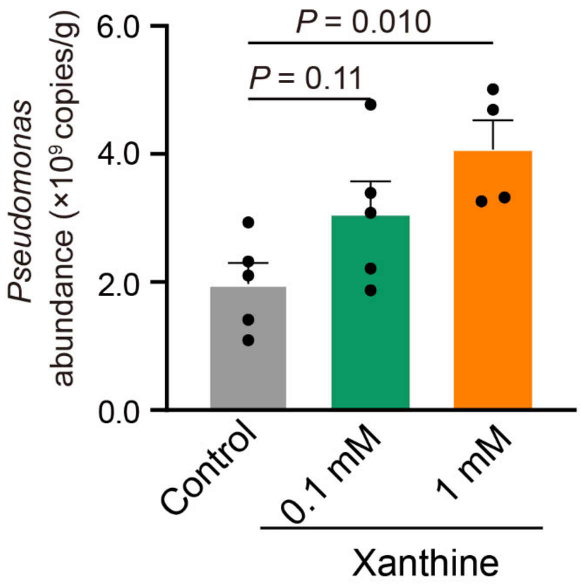

Supplementary Figure 16. Root *Pseudomonas* abundance upon application of xanthine. Root samples of two levels of xanthine (0.1 mM and 1 mM) and a control group (0 mM) were measured by qPCR with lineage-specific primers. Significance was determined with two-sided Student's *t*-test. For each data point, mean ± SEM, $n = 5$ biological replicates except for 1 mM xanthine treatment ($n = 4$ biological replicates). P values are determined by two-sided Student's *t*-test. Source data are provided as a Source Data file.

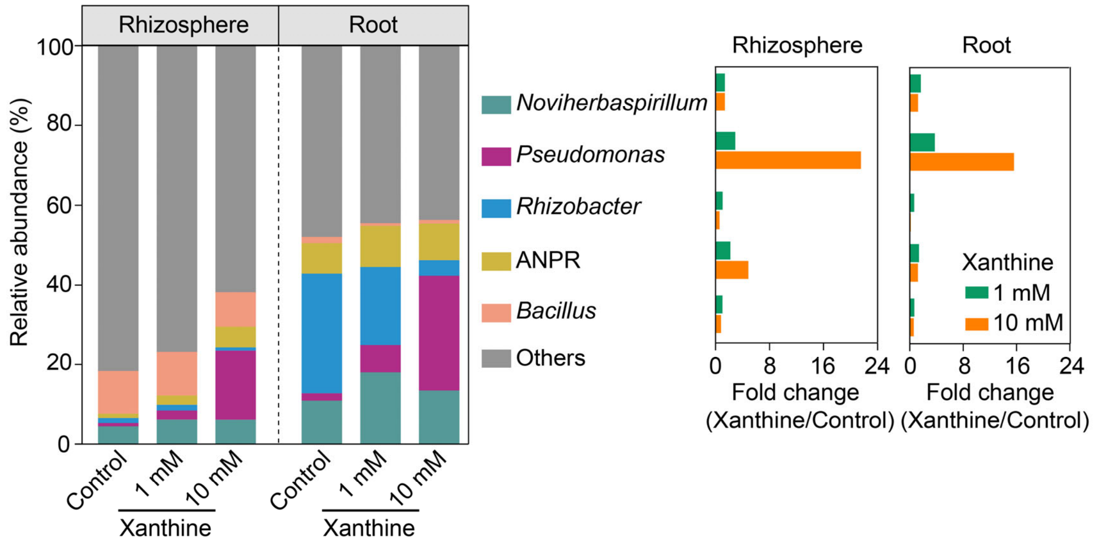

Supplementary Figure 17. Relative abundance of bacteria at the genus level in rhizosphere soil and root after application of xanthine. The top five most abundant genera in control and xanthine addition groups (left), and their fold change between xanthine addition group and control (right). The relative abundance of each genus is the average values of six biological replicates. ANPR: Allorhizobium-Neorhizobium-Pararhizobium-Rhizobium. Source data are provided as a Source Data file.

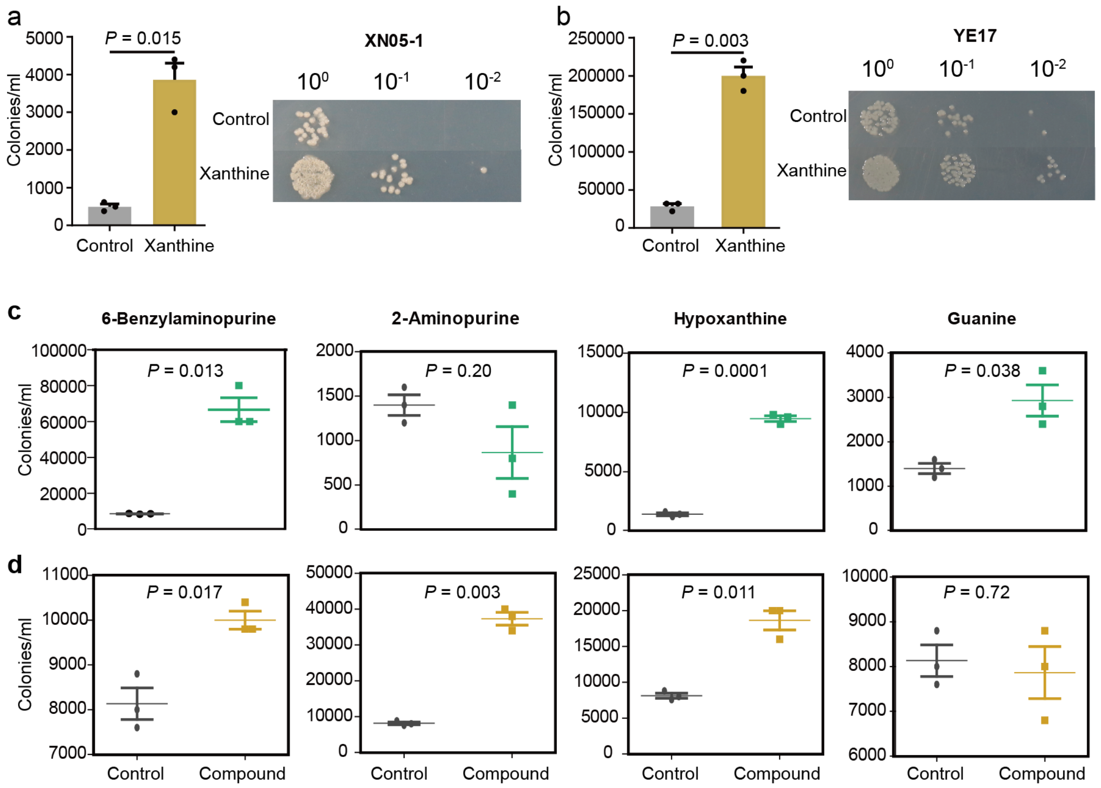

Supplementary Figure 18. The chemotaxis of strain XN05-1 and YE17 toward purines or its derivatives. a The chemotaxis of strain XN05-1 toward to xanthine. **b** The chemotaxis of strain YE17 toward to xanthine. **c** The chemotaxis of strain XN05-1 toward to other purines or its derivatives. **d** The chemotaxis of strain YE17 toward to other purines or its derivatives. P values are determined by two-sided Student's t-test. For each data point, mean ± SEM, $n = 3$ biological replicates. Source data are provided as a Source Data file.

Supplementary Table 1. PERMANOVA analysis of the microbial community composition of compartment time point and treatment based on Bray-Curtis distance metric at the OTU level.

Data used	Characteristics	SumsOfSqs	MeanSqs	F_Model	R2 (%)	P_value
Whole data	Compartment	13.41189	6.71	81.66	53.843	0.001
	Time point	0.89096	0.44548	2.60	3.577	0.028
	Treatment	0.67041	0.67041	3.90	2.691	0.019

Supplementary Table 2. The information of all *Pseudomonas* strains isolated in this study. Values in bold represent the highest similarities between isolates and OTUs.

**Strains**	**Top-hit taxon based on 16S rRNA gene**	**Similarity (%) with OTU 4227**	**Similarity (%) with OTU 2336**
CL07	*Pseudomonas alcaligenes*	96.56	95.5
YE35	*Pseudomonas alcaligenes*	95.86	95.86
YL16	*Pseudomonas alcaligenes*	95.86	95.86
YE17	*Pseudomonas frederiksbergensis*	94.44	**99.21**
YL01	*Pseudomonas frederiksbergensis*	94.44	**99.21**
YL02	*Pseudomonas frederiksbergensis*	94.44	**99.21**
RL01	*Pseudomonas frederiksbergensis*	94.44	98.94
RL02	*Pseudomonas frederiksbergensis*	94.44	98.94
YE23	*Pseudomonas frederiksbergensis*	94.44	98.94
YL08	*Pseudomonas frederiksbergensis*	94.44	98.94
LJ18	*Pseudomonas geniculata*	88.92	88.71
LJ20	*Pseudomonas geniculata*	88.92	88.71
CL10	*Pseudomonas indica*	93.93	94.72
CS07	*Pseudomonas kunmingensis*	95.77	96.3
RE01	*Pseudomonas kunmingensis*	95.77	96.3
RE04	*Pseudomonas kunmingensis*	95.77	96.3
YE21	*Pseudomonas kunmingensis*	95.77	96.3
YE22	*Pseudomonas kunmingensis*	95.77	96.3
YL12	*Pseudomonas kunmingensis*	95.77	96.3
LJ02	*Pseudomonas mosselii*	96.83	97.88
CS03	*Pseudomonas oleovorans*	**97.88**	96.83
XN03	*Pseudomonas oleovorans*	**97.88**	96.83
CS12	*Pseudomonas songnenensis*	95.89	95.89
RE09	*Pseudomonas songnenensis*	95.5	95.77
YL05	*Pseudomonas songnenensis*	94.97	95.77
YL09	*Pseudomonas songnenensis*	95.24	95.5
YS01	*Pseudomonas songnenensis*	95.24	95.5
XN05-1	*Pseudomonas stutzeri*	95.77	96.3
XN05-2	*Pseudomonas stutzeri*	95.77	96.3
CS05	*Pseudomonas xanthomarina*	95.77	96.3
CY09	*Pseudomonas xanthomarina*	95.77	96.3

RL08	*Pseudomonas xanthomarina*	96.83	95.77
RL15	*Pseudomonas xanthomarina*	95.77	96.03
YY08	*Pseudomonas xanthomarina*	95.77	96.3

Supplementary Table 3. The numbers of total gene, differentially expressed gene (DEG), upregulated and downregulated genes in bacterial chemotaxis and flagellar assembly subcategories determined by metatranscriptomic data.

	**Total genes**	**DEGs**	**Upregulated genes**	**Downregulated genes**
**Bacterial chemotaxis**	672	395	329	66
**Flagellar assembly**	1018	729	654	75

Supplementary Table 4. The top 10 most significantly enriched root exudates in two salt treatments. $log_{10}FC$ (100) represents the log_{10}-fold change of compounds between 100 mM NaCl treated and control plants. $log_{10}FC$ (200) represents the log_{10}-fold change of metabolites between 200 mM NaCl treated and control plants. *P* values are determined by two-sided Student's *t*-test (*P* values were shown in brackets).

Compounds	Category	$log_{10}FC$ (100)	$log_{10}FC$ (200)
N-Cyclohexylformamide	Alkaloids	6.46 (0.005)	6.64 (6.2×10^{-5})
Xanthine	Nucleotides and derivatives	4.85 (0.032)	6.07 (0.0007)
2,4,5-Trimethoxybenzoic acid	Phenolic acids	5.04 (0.0097)	5.31 (0.002)
N-Ethylmaleimide	Amino acids and derivatives	5.30 (0.0008)	5.30 (0.001)
2-Acetyl-5-methylfuran	Others	5.06 (0.0013)	5.16 (0.0003)
4-Hydroxybenzoylmalic acid	Phenolic acids	4.24 (0.012)	5.15 (0.001)
1,2-Decanediol	Others	4.87 (0.006)	5.13 (0.002)
5-Aminovaleric acid	Organic acids	4.77 (0.015)	5.05 (0.0002)
2-Isopropylmalic Acid	Organic acids	4.79 (0.032)	5.05 (0.003)
L-Valyl-L-Phenylalanine	Amino acids and derivatives	4.24 (0.034)	5.02 (0.005)

Supplementary Table 5. The top 10 most abundant compounds of nucleotides and derivatives category. $log_{10}FC$ (100) represents the log_{10}-fold change of compounds between 100 mM NaCl treated and control plants. $log_{10}FC$ (200) represents the log_{10}-fold change of metabolites between 200 mM NaCl treated and control plants. *P* values are determined by two-sided Student's *t*-test (*P* values were shown in brackets).

**Compounds**	**$log_{10}FC$ (100)**	**$log_{10}FC$ (200)**
Xanthine	4.85 (0.032)	6.07 (0.0007)
**6-Benzylaminopurine**	**3.33 (0.034)**	**3.96 (0.008)**
**2-Aminopurine**	**3.90 (0.022)**	**3.74 (0.027)**
5-Methyluridine	3.16 (0.003)	3.40 (2.7×10^{-5})
Uridine 5'-monophosphate	4.04 (0.041)	3.35 (0.022)
**Hypoxanthine**	**0.95 (0.0001)**	**2.07 (0.001)**
**Guanine**	**1.16 (0.001)**	**2.06 (0.001)**
β-Pseudouridine	1.29 (6.8×10^{-5})	1.88 (0.002)
8-Azaguanine	0.78 (0.006)	1.88 (0.004)
Cytarabine	1.58 (0.005)	1.59 (0.006)

Supplementary Table 6. Primers used in this study.

Name	Sequences (5'-3')	Annealing temperature	Usage	Reference
799F	AACMGGATTAGATACCCKG	55	16S rRNA gene amplicon sequencing	(1)
1193R	ACGTCATCCCCACCTTCC			(2)
Eub338	ACTCCTACGGGAGGCAGCAG	55	qPCR for bacteria	(3)
Eub518	ATTACCGCGGCTGCTGG			
Ps-for	GAGTTTGATCCTGGCTCAG	50	qPCR for *Pseudomonas*	(4)
Ps-rev	CCTTCCTCCCAACTT			
27F	AGAGTTTGATCCTGGCTCAG	55	Isolates identification	(5)
1492R	GGTTACCTTGTTACGACTT			
XN05-1-cheW1-UF	gagctcggtacccggggatccAGCCCTTCGAGTGTTTGCTG		UF/UR and DF/DR are for PCR amplification of the upstream and downstream homologous arms of strain XN05-1 *cheW1*, respectively	This study
XN05-1-cheW1-UR	TGCGCAGACGTCATCTTCATA			
XN05-1-cheW1-DF	atgaagatgacgtctgcgcaCCGAGCTTGAGAGCATCTGATAC			
XN05-1-cheW1-DR	acgacggccagtgccaagcttCGCCCTCCTCGTTCCGAA			
XN05-1-cheW2-UF	gagctcggtacccggggatccAACGGTCTGGGGCTGGTG		UF/UR and DF/DR are for PCR amplification of the upstream and downstream homologous arms of strain XN05-1 *cheW2*, respectively	This study
XN05-1-cheW2-UR	ttgtgcggctttgGTAAGGACGGTGCGACTCATG			
XN05-1-cheW2-DF	tccttacCAAAGCCGCACAAGCGCT			
XN05-1-cheW2-DR	acgacggccagtgccaagcttCAGATGCTCTCAAGCTCGGC			
XN05-1-cheW3-UF	gagctcggtacccggggatccAAAGCGCGAAAATCACCGA		UF/UR and DF/DR are for PCR	This study

XN05-1-cheW3-UR	attgagGCTGTGCTCGGTATTGGCG		amplification of the upstream and downstream homologous arms of strain XN05-1 *cheW3*, respectively	
XN05-1-cheW3-DF	ataccgagcacagcCTCAATCGCGCCATGGCG			
XN05-1-cheW3-DR	acgacggccagtgccaagcttGCAGGTGCTGCAACAGACG			
YE17-cheW1-UF	gagctcggtacccggggatccATTGCAGGGCCTGGTGACA		UF/UR and DF/DR are for PCR amplification of the upstream and downstream homologous arms of strain YE17 *cheW1*, respectively	This study
YE17-cheW1-UR	ttccgaAGACGACGCCTTATCATTCATACC			
YE17-cheW1-DF	ataaggcgtcgtctTCGGAACTGGAGAGTATCTGATTTG			
YE17-cheW1-DR	acgacggccagtgccaagcttTTGACGTCATAGGATTTCGAAGC			
YE17-cheW2-UF	gagctcggtacccggggatccAGAGCGGCCTGGGCCTGG		UF/UR and DF/DR are for PCR amplification of the upstream and downstream homologous arms of strain YE17 *cheW2*, respectively	This study
YE17-cheW2-UR	ttccggaccggTGTCTTGATCGGGCGGTTC			
YE17-cheW2-DF	atcaagacaCCGGTCCGGAAACCGAAA			
YE17-cheW2-DR	acgacggccagtgccaagcttACTCTCCAGTTCCGACCACTCTT			
YE17-cheW3-UF	gagctcggtacccggggatccCGATCGAAGCAGCCCGCG		UF/UR and DF/DR are for PCR amplification of the upstream and downstream homologous arms of strain YE17 *cheW3*, respectively	This study
YE17-cheW3-UR	atgagtctggcgcttccaGTGGTCGCGATGGCGCCC			
YE17-cheW3-DF	acTGGAAGCGCCAGACTCATG			
YE17-cheW3-DR	acgacggccagtgccaagcttCCCTGCTGGCTGCCGGTG			

References

1. Chelius, M. K., Triplett, E. W. The diversity of archaea and bacteria in association with the roots of *Zea mays* L. *Microbial Ecology*. **41**, 252-263 (2001).
2. Bodenhausen, N., Horton, M. W., Bergelson, J. Bacterial communities associated with the leaves and the roots of *Arabidopsis thaliana*. *PLoS One*. **8**, e56329 (2013).
3. Muyzer, G., de Waal, E. C., Uitterlinden, A. G. Profiling of complex microbial populations by denaturing gradient gel electrophoresis analysis of polymerase chain reaction-amplified genes coding for 16S rRNA. *Applied and Environmental Microbiology*. **59**, 695-700 (1993).
4. Johnsen, K., Enger, Ø., Jacobsen, C. S., Thirup, L., Torsvik, V. Quantitative selective PCR of 16S ribosomal DNA correlates well with selective agar plating in describing population dynamics of indigenous *Pseudomonas* spp. in soil hot spots. *Applied and Environmental Microbiology*. **65**, 1786-1788 (1999).
5. Weisburg, W. G., Barns, S. M., Pelletier, D. A., Lane, D. J. 16S ribosomal DNA amplification for phylogenetic study. *Journal of Bacteriology*. **173**, 697-703 (1991).
