# Supplementary material for: Purines enrich root-associated Pseudomonas and improve wild soybean growth under salt stress
Source: Nat Commun. 2024 Apr 25;15:3520. doi: 10.1038/s41467-024-47773-9 (PMC11045775; doi:10.1038/s41467-024-47773-9)
Supplement: Supplementary file 2 — Description of Additional Supplementary Files [file 41467_2024_47773_MOESM2_ESM.pdf]

Description of Additional Supplementary Files

Supplementary Data 1.
Enriched or depleted genera in root, rhizosphere and bulk soil of salt-treated groups after 14 days of salt stress. The log_2 fold enrichment (positive value) or depletion (negative value) for each genus under salt stress and their relative abundance in control and salt treatments were shown. The value of zero indicate the genus does not have significant enrichment or depletion for that sample.

Supplementary Data 2.
Genes involved in chemotaxis and flagellar assembly of *P. stutzeri* XN05-1 and *P. frederiksbergensis* YE17.

Supplementary Data 3.
Summary of samples used for multi-omics. The detailed information was shown for all samples.
