# Supplementary material for: Purines enrich root-associated Pseudomonas and improve wild soybean growth under salt stress
Source: Nat Commun. 2024 Apr 25;15:3520. doi: 10.1038/s41467-024-47773-9 (PMC11045775; doi:10.1038/s41467-024-47773-9)
Supplement: Supplementary file 4 — Reporting Summary [file 41467_2024_47773_MOESM4_ESM.pdf]

nature portfolio

Corresponding author(s): Dr. Yanfen Zheng and Prof. Cheng-Sheng Zhang

Last updated by author(s): Apr 5, 2024

Reporting Summary

Nature Portfolio wishes to improve the reproducibility of the work that we publish. This form provides structure for consistency and transparency in reporting. For further information on Nature Portfolio policies, see our Editorial Policies and the Editorial Policy Checklist.

Statistics

For all statistical analyses, confirm that the following items are present in the figure legend, table legend, main text, or Methods section.

n/a	Confirmed	
☐	☒	The exact sample size (*n*) for each experimental group/condition, given as a discrete number and unit of measurement
☐	☒	A statement on whether measurements were taken from distinct samples or whether the same sample was measured repeatedly
☐	☒	The statistical test(s) used AND whether they are one- or two-sided<br>*Only common tests should be described solely by name; describe more complex techniques in the Methods section.*
☒	☐	A description of all covariates tested
☐	☒	A description of any assumptions or corrections, such as tests of normality and adjustment for multiple comparisons
☐	☒	A full description of the statistical parameters including central tendency (e.g. means) or other basic estimates (e.g. regression coefficient) AND variation (e.g. standard deviation) or associated estimates of uncertainty (e.g. confidence intervals)
☐	☒	For null hypothesis testing, the test statistic (e.g. *F*, *t*, *r*) with confidence intervals, effect sizes, degrees of freedom and *P* value noted<br>*Give P values as exact values whenever suitable.*
☒	☐	For Bayesian analysis, information on the choice of priors and Markov chain Monte Carlo settings
☒	☐	For hierarchical and complex designs, identification of the appropriate level for tests and full reporting of outcomes
☐	☒	Estimates of effect sizes (e.g. Cohen's *d*, Pearson's *r*), indicating how they were calculated

Our web collection on statistics for biologists contains articles on many of the points above.

Software and code

Policy information about availability of computer code

Data collection	Illumina MiSeq, Illumina HiSeq X-Ten, Illumina HiSeq 2500, PacBio Sequel
Data analysis	The software packages used for 16S rRNA gene amplicon data analysis were Flash v1.2.11, Qiime v1.9.1, Uparse v7.0.1.90, RDP Classifier v2.11, Usearch v7.0 and Mothur v1.30.2. The software packages used for metagenome and metatranscriptome data analyses were Fastp v0.20.0, Megahit v1.1.2, MetaGene, CD-HIT v4.8.1, BLASTp v2.3.0, Diamond v0.8.35, METAProdigal v2.6.3, bbmap, samtools, metabat2 and Salmon v1.1.0. The software packages used for whole genome sequencing data were SMRT Link v5.0.1 and blast. R v3.5.3, GraphPad Prism 9, TBtools and MEGA (v7.0) were applied for plotting. The scripts used in this study are available at GitHub (https://github.com/helloacc/wildsoybean_microbiome).

For manuscripts utilizing custom algorithms or software that are central to the research but not yet described in published literature, software must be made available to editors and reviewers. We strongly encourage code deposition in a community repository (e.g. GitHub). See the Nature Portfolio guidelines for submitting code & software for further information.

Data

Policy information about availability of data

All manuscripts must include a data availability statement. This statement should provide the following information, where applicable:

- Accession codes, unique identifiers, or web links for publicly available datasets
- A description of any restrictions on data availability
- For clinical datasets or third party data, please ensure that the statement adheres to our policy

Raw 16S rRNA gene amplicon sequence and metagenomic data derived from salt stress experiments are publicly available under NCBI BioProject number PRJNA890906 [https://www.ncbi.nlm.nih.gov/bioproject/?term=PRJNA890906]. Metatranscriptomic data has been deposited in the Sequence Read Archive under BioProject number PRJNA989423 [https://www.ncbi.nlm.nih.gov/bioproject/?term=PRJNA989423]. All 16S rRNA sequence data derived from xanthine application experiments are publicly available under NCBI BioProject number PRJNA1064605 [https://www.ncbi.nlm.nih.gov/bioproject/?term=PRJNA1064605]. The genomic data for strains XN05-1 and YE17 are publicly available under NCBI BioProject number PRJNA1061746 [https://www.ncbi.nlm.nih.gov/bioproject/?term=PRJNA1061746]. The SILVA database is available at https://www.arb-silva.de/. The NCBI-nr database is available at https://www.ncbi.nlm.nih.gov/public/. The KEGG database is available at http://www.genome.jp/kegg/. The eggNOG database is available at http://eggnog6.embl.de/#/app/home. Source data are provided with this paper.

Research involving human participants, their data, or biological material

Policy information about studies with human participants or human data. See also policy information about sex, gender (identity/presentation), and sexual orientation and race, ethnicity and racism.

Reporting on sex and gender	Not applicable
Reporting on race, ethnicity, or other socially relevant groupings	Not applicable
Population characteristics	Not applicable
Recruitment	Not applicable
Ethics oversight	Not applicable

Note that full information on the approval of the study protocol must also be provided in the manuscript.

Field-specific reporting

Please select the one below that is the best fit for your research. If you are not sure, read the appropriate sections before making your selection.

☒ Life sciences ☐ Behavioural & social sciences ☐ Ecological, evolutionary & environmental sciences

For a reference copy of the document with all sections, see nature.com/documents/nr-reporting-summary-flat.pdf

Life sciences study design

All studies must disclose on these points even when the disclosure is negative.

Sample size	Sample size for metageonmic (n=4) and metatranscriptomic (n=3) sequencings were based on a previous study (Xu et al., 2018, PNAS). For other experiments, no statistical methods used to predetermine sample size. The samples size was determined by the requirement for statistical analyses and shown in the relavant figure legends.
Data exclusions	In the phenotyping experiment, seedlings with poor germination were excluded. No other data were excluded from analyses.
Replication	The phenotyping experiments and quantitative chemotaxis assays were conducted independently at least three times. They were replicated successfully. Samples for amplicon, metagenomic and metatranscriptomic sequencing were collected independently, and the main finding related to taxonomic analyses were replicated successfully. For other experiments, the number of replication (at least three repeats) was shown in the relavant figure legends.
Randomization	Wild soybean seedlings with the same genotype were randomly allocated to experimental groups. For other experiments, groups were allocated based on the treatment, thus randomization was not applicable.
Blinding	Sampling, phenotyping experiment and qPCR for Pseudomonas were performed by two assistants who did not know the experimental design. DNA/RNA extractions and sequencing were conducted by unfamiliar technicians from sequencing company. Blinding was not applicable for other analyses and experiments.

Reporting for specific materials, systems and methods

We require information from authors about some types of materials, experimental systems and methods used in many studies. Here, indicate whether each material, system or method listed is relevant to your study. If you are not sure if a list item applies to your research, read the appropriate section before selecting a response.

Materials & experimental systems

n/a	Involved in the study	
☒	☐	Antibodies
☒	☐	Eukaryotic cell lines
☒	☐	Palaeontology and archaeology
☒	☐	Animals and other organisms
☒	☐	Clinical data
☒	☐	Dual use research of concern
☐	☒	Plants

Methods

n/a	Involved in the study	
☒	☐	ChIP-seq
☒	☐	Flow cytometry
☒	☐	MRI-based neuroimaging

Dual use research of concern

Policy information about dual use research of concern

Hazards

Could the accidental, deliberate or reckless misuse of agents or technologies generated in the work, or the application of information presented in the manuscript, pose a threat to:

No	Yes	
☒	☐	Public health
☒	☐	National security
☒	☐	Crops and/or livestock
☒	☐	Ecosystems
☒	☐	Any other significant area

Experiments of concern

Does the work involve any of these experiments of concern:

No	Yes	
☒	☐	Demonstrate how to render a vaccine ineffective
☒	☐	Confer resistance to therapeutically useful antibiotics or antiviral agents
☒	☐	Enhance the virulence of a pathogen or render a nonpathogen virulent
☒	☐	Increase transmissibility of a pathogen
☒	☐	Alter the host range of a pathogen
☒	☐	Enable evasion of diagnostic/detection modalities
☒	☐	Enable the weaponization of a biological agent or toxin
☒	☐	Any other potentially harmful combination of experiments and agents
